# Integrating Network Pharmacology and Experimental Models to Investigate the Mechanism of Huanglian Jiedu Decoction on Inflammatory Injury Induced by Cerebral Ischemia

**DOI:** 10.1155/2021/2135394

**Published:** 2021-01-13

**Authors:** HuiMin Li, Teng Lv, Bin Wang, Min Li, JiPing Liu, Chuan Wang, ZhiShu Tang

**Affiliations:** Shaanxi University of Chinese Medicine, Xianyang 712046, China

## Abstract

Unlike single-target Western medicines, traditional Chinese medicines (TCMs) exhibit diverse curative effects against multiple diseases through their “multicomponent” and “multitarget” manifestations. However, the material basis of the major therapeutic diseases and TCM underlying molecular mechanisms remain to be challenged. In the current study, we applied, for the first time, an integrated strategy that combines network pharmacology and experimental evaluation and explored and demonstrated the underlying possible mechanisms of a classic TCM formula, Huanglian Jiedu Decoction (HLJD), in the treatment of cerebral ischemia. First, the herb compound, protein compound, and GO-BP and KEGG pathways were constructed to predict the material basis of HLJD in the treatment of cerebral ischemia and explore the underlying molecular mechanisms. Network pharmacology analysis showed that HLJD treats cerebral ischemia mainly through its anti-inflammatory effect. We used molecular docking to verify that HLJD components have good binding activities to the arachidonic acid pathway enzymes, cyclooxylipase-2 (COX-2) and 5-lipoxygenase (5-LOX). Next, based on the prediction by the network pharmacology analysis, the rat model of middle cerebral artery occlusion (MCAO) was established to verify the efficacy of HLJD. The results showed that HLJD reduces the degree of brain injury in MCAO rats, probably by inhibiting the expression of the 5-LOX pathway and inflammatory response. In conclusion, our study demonstrates the effectiveness of integrating network pharmacology with an experimental study for material basis of the major therapeutic diseases and the underlying molecular mechanisms of TCM.

## 1. Introduction

Huanglian Jiedu Decoction (HLJD), originally recorded in the Secret of Waitai, is one of the representative prescriptions of heat-clearing and detoxifying drugs, which is composed of *Huanglian* (*Coptis chinensis Franch*), *Huangqin* (*Scutellaria baicalensis Georgi*), *Huangbo* (*Phellodendron amurense Rupr*), and *Zhizi* (*Gardenia jasminoides Ellis*) [1]. It has a detoxification effect, clears heat, and resolves dampness. The main treatment is Sanjiao heat Sheng, and the symptoms are associated with strong heat, dry mouth, incoherent language, sleeplessness, fever, blood vomiting, carbuncles, a red tongue, yellow moss, and increased pulse [2]. HLJD is clinically used for the treatment of infectious, and cerebrovascular diseases and diabetes [3]. Its main pharmacological effects are anti-inflammatory, antipyretic, antibacterial, and antiviral. It also prevents cerebral ischemia and has an antithrombosis effect [4]. There are many reports on the pharmacological effects of HLJD, but its active components and mechanisms of action are not well known, which largely limits the application of this drug and hinder the inheritance and development of national and traditional drugs.

Modern medicine believes that cerebral ischemia is due to the narrowing or blockage of cerebral arteries, resulting in ischemic and hypoxic injuries that are caused by insufficient blood supply to the brain. The timely recovery of the brain blood supply is the best treatment. However, subsequent blood perfusion will bring corresponding problems, including secondary brain injury that is caused by inflammation. The prevention of cerebral ischemia-reperfusion injury is also one of the research hotspots [5]. The theory of traditional Chinese medicine advocates the prevention of “toxic damage to the brain and collaterals.” In recent years, HLJD has been widely used in the treatment of cerebral ischemia under the guidance of the theory of traditional Chinese medicine [6]. It is pointed out that the effect of HLJD on cerebral ischemia may be related to its anti-inflammatory effect.

**Figure 1:**
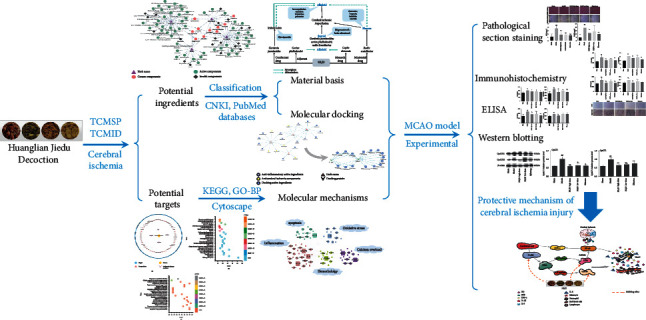
The detailed flowchart of the current study.

The mode of multiple components, targets, and pathways is a prominent feature of TCM. It is believed that a TCM formulae possess numerous chemical components acting on multiple targets and diseases [7]. In order to comprehensively evaluate the pharmacological effects of TCM, network pharmacology has been introduced to explore the molecular mechanism of TCM in recent years [8, 9]. Based on network interaction to study the basic biological knowledge of TCM can provide a deep insight or scientific evidence for the discovery of TCM and help us to clarify the pharmacological mechanism of active ingredients of TCM at the level of biomolecule [10]. Combined with pharmacology and pharmacodynamics, it has been successfully applied to explain the mechanism of TCM at the molecular network level [11]. The molecular docking approach has become an increasingly important tool in pharmaceutical research and can be used to model the interaction between a small molecule and a protein at the atomic level, which enables the identification of potential drug targets, as well as the ability to characterize the behavior of small molecules in the binding site of target proteins [12]. On this basis, we applied, for the first time, a network pharmacology approach to predicting HLJD major potential targets and related pathways, and explored the underlying molecular mechanisms. Meanwhile, a rat model of cerebral ischemia/reperfusion injury models were established to validate HLJD curative role as predicted by the network pharmacology analysis and molecular docking. The detailed procedures can be seen in Figure 1.

## 2. Materials and Methods

### 2.1. Screening of Bioactive Components

All of the constituent data for HLJD were obtained from the Traditional Chinese Medicine Systems Pharmacology Database and Analysis Platform (TCMSP, http://lsp.nwu.edu.cn/tcmsp.php) [13]. The bioactive ingredients of HLJD were obtained from PubMed (http://www.ncbi.nlm`ih.gov/pubmed), CNKI (https://www.cnki.net/), and Springer (https://link.springer.com/) databases. The active components and targets were further identified using the parameters of the oral bioavailability (OB) threshold that was ≥30% and a drug likeness (DL) that was ≥0.18. FDA-approved targets for anticerebral ischemic injury drugs were collected from the Therapeutic Target Database (TTD) (http://bidd.nus.edu.sg/group/cjttd/), DrugBank (https://www.drugbank.ca/), GeneCards (https://www.genecards.org/), and DisGeNET (http://www.disgenet.org/) databases. The targets obtained for HLJD and the prediction targets for cerebral ischemia were mapped into Venny 2.1.0 (https://bioinfogp.cnb.csic.es/tools/venny/index.html) to obtain the anti-ischemic targets for HLJD.

#### 2.1.1. Network Construction and Analysis

The network was built using the Cytoscape 3.4.0 software and the network analyzer plugin. After the analysis of the compound-target network and target-pathway network, it was concluded that HLJD had anticerebral ischemic injury effects.

#### 2.1.2. Identification of Potential Pathways

To elucidate the pathways targeted by the compounds and any gene-associated diseases, the identified genes were further analyzed using Integrated Discovery (David, https://david.ncifcrf.gov/). A threshold count ≥10 and EASE scores ≤0.05 were chosen for functional annotation clustering.

### 2.2. Molecular Docking

The effective components of HLJD were screened by CNKI, PubMed, and Springer databases, and the 3D crystal structure of key target proteins was downloaded by the RCSB PDB database (http://www.rcsb.org/pdb/home/home.do); the molecular structure was downloaded by TCMSP and PubChem (https://pubchem.ncbi.nlm.nih.gov/). Proteins preparation and subsequent molecular docking were performed using Discovery Studio 2.5 and AutoDock [14].

### 2.3. Preparation of HLJD Aqueous Extract

HLJD consisted of 900 g of *Huanglian*, 600 g of *Huangbo*, 600 g of *Huangqin*, and 900 g of *Zhizi*. All crude herbs were provided from the dispensary of Chinese Medicine, the University of Shaanxi University of Chinese Medicine. All crude herbs were mixed and boiled in two batches. The first batch had 10 times as much water and was filtered for 1 h, and the second batch had 8 times as much water and was filtered for 1 h. The filtrates were combined two times, and the concentrated paste rate was 28.4%. The freeze-dried water extract can produce 853 g dry powder, which is dissolved in water by stirred at 37°C for 1 h, filtered, disinfected, and refrigerated at 4°C ready for use. HPLC was used to determine baicalin, berberine hydrochloride, and gardenoside in HLJD extract. Baicalin, geniposide, and berberine were purchased from Solarbio Bio Inc. Beijing, content ≥98%.

### 2.4. Middle Cerebral Artery Occlusion (MCAO) Model

Adult male Sprague-Dawley rats (250∼320 g) were obtained from the Experimental Animal Center of Xi'an Jiaotong University (Shaanxi, China). The permission number was SCXK (Shaanxi) 2012-003. The animal welfare and experimental procedures were conducted in strict accordance with the guidelines for the care and use of experimental animals and the ethics of Shaanxi University of Chinese Medicine. All animals had free access to standard diet and unlimited water. The rats were randomly divided into 5 groups: the sham operation control, the model, HLJD high-dose (12.5 g/kg), HLJD low-dose (6.25 g/kg), and the positive drug groups (edaravone 6.25 × 10^−3 ^g/kg, zileuton).

A modified Longa method was applied to prepare the model of middle cerebral artery embolism in rats [15, 16]. Rats were fixed on their back after anesthesia. A midline incision was made at the neck to separate the common carotid artery, the external carotid artery, and the internal carotid artery on the left side. Arteries were marked by ligatures on the external carotid artery and the proximal end of the common carotid artery. A 0.285 mm nylon thread was inserted from the distal end of the common carotid artery to the internal carotid artery, about 18.0 ± 0.5 mm in depth to the anterior cerebral artery. The nylon thread was not applied in the sham operation group. After operation 24 h, the brain and the blood were taken.

### 2.5. Enzyme-Linked Immunosorbent Assay (ELISA)

The concentrations of neuron-specific enolase (NSE) and activation marker protein (S100 B) in serum were determined using ELISA kit according to the manufacturer's instructions. Inflammatory factors and 5-LOX pathway proteins in brain homogenate were determined using ELISA kit according to the manufacturer's instructions. All ELISA kits were purchased from Wuhan Genmei Biotechnology Co. Ltd.

### 2.6. Pathological Section Staining

The brain was decapitated 24 hours after operation, embedded in paraffin and preserved at 4°C. Haematoxylin-eosin (H&E) staining and Nissl's Staining were performed.

### 2.7. Immunohistochemistry Analysis

The immunohistochemistry assay for nuclear factor-*κ*Bp65 (NF-*κ*Bp65) in brain tissue was performed according to a previously described protocol [17]. NF-kBp65 immunohistochemistry kit was purchased from Wuhan Genmei Biotechnology Co. Ltd.

### 2.8. Western Blotting (WB)

Protein samples were separated by 10% SDS/PAGE and transferred to PVDF membrane. The membrane was blocked with 1% BSA in TBST, incubated with antibodies in TBST, and developed in electrochemiluminescence (ECL) plus western blotting detection reagents (Abeam, Cambridge, USA). The chemiluminescence signal was analyzed with MultiGauge.

### 2.9. Statistical Analyses

All the data were statistically analyzed by the SPSS 24 software and expressed as mean ± SD. Single-factor analysis of variance (ANOVA) was used to compare the differences among the three groups, and *p* < 0.05 was defined as a statistically significant difference.

## 3. Results

### 3.1. Identification of HLJD Bioactive Compounds and Targets

**Figure 2:**
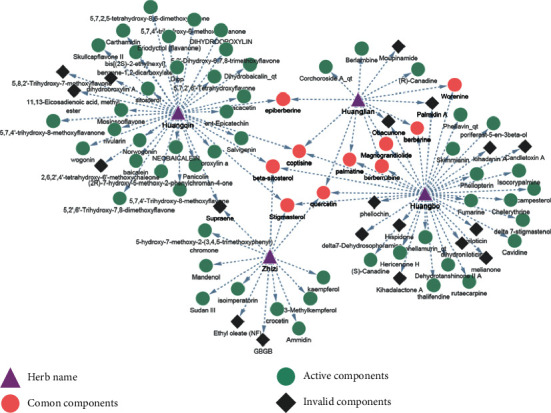
Diagram of the composition of HLJD.

**Figure 3:**
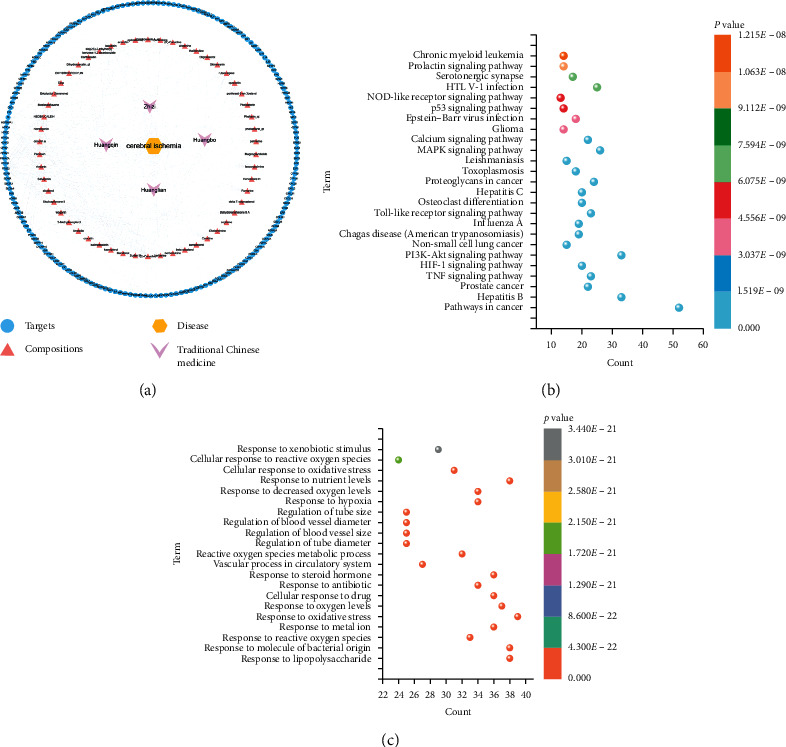
(a) “Drug-component-target-disease” network diagram; (b) KEGG enrichment analysis; (c) GO-BP enrichment analysis.

A total of 102 active components were screened from the TCMSP and TCMID databases, including 37 components of *Cortex phellodendri*, 14 components of *Coptis chinensis*, 36 components of *Scutellaria baicalensis*, and 15 components of *Gardenia jasminoides* (Figure 2). A total of 180 common target genes were screened by DisGeNET, CTD, NCBI Gene, OMIM, DrugBank, PharmGKB, and GeneCards search for the intersection of disease-related target genes and HLJD active components. The data were imported into the Cytoscape software to construct a “drug-component-target-disease” map (Figure 3(a)). HLJD key targets and active components against cerebral ischemia were screened by degree and BC. The main active components were quercetin, kaempferol, wogonin, *β*-sitosterol, isocorypalmine, and baicalein. Their main targets are PTGS2, PTGS1, HSP90AA1, and PRKACA.

### 3.2. GO and KEGG Enrichment Analysis for the Target Proteins

A total of 7112 enrichment results were obtained by GO-BP, and the enrichment pathways are shown in Figure 3(c). The pathways are mainly involved in the response to reactive oxygen species, coping peptides, oxidative stress, cell response to drugs, metabolism of reactive oxygen species, and response to steroids.

The KEGG pathway of HLJD intersection and cerebral ischemia genes were enriched by the DAVID database, and the first 20 pathways are listed in Figure 3(b) and involve the PI3K-Akt and the MAPK signaling pathways, the HTLV-I infection pathway, the tumor necrosis factor signaling pathway, the calcium signaling pathway, the focal adhesion, and the cAMP signaling pathway.

According to the analysis of the enrichment results of GO and KEGG pathways, it was found that many cancer pathways and inflammation-related pathways were involved, such as inflammatory response to antigenic stimulation, dopamine receptor signaling pathway, response to calcium ion, cell response to calcium ion, regulation of glial cell differentiation, the MAPK signaling pathway, the tumor necrosis factor signaling pathway, and the calcium signaling pathway that is related to cerebral ischemic injury. Combined with the results of target screening, HLJD can be mainly used to treat cerebral ischemic injury by inhibiting the occurrence of inflammation. 5-LOX and COX-2, which are metabolites of arachidonic acid, are common inflammatory targets of HLJD. To verify whether HLJD can act on these two proteins, we verified the bioactivity of HLJD to these proteins by molecular docking.

### 3.3. Molecular Docking

**Figure 4:**
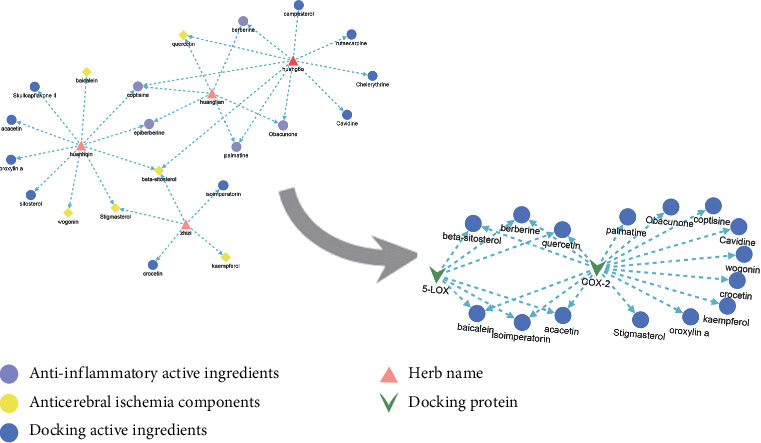
Active ingredients with anti-inflammatory effects in HLJD.

Through CNKI, PubMed, and Springer databases for rescreening, screening can identify the anti-inflammatory effect of a total of 21 active components. The related data are shown in Figure 4.

After molecular docking by AutoDock, the docking information of HLJD active components with COX-2 and 5-LOX was obtained and included in the free energy evaluation score and inhibition constant (Ki) (Table 1). The docked molecules were sorted according to the free energy by function analysis, and the conformation with the lowest free energy was selected as optimal. Compared with the positive control and screening results, it was found that HLJD anti-inflammatory components could act on two key inflammatory proteins, COX-2 and 5-LOX, of which 19 HLJD components had anti-inflammatory effects and were effective against cerebral ischemia. Therefore, it is speculated that HLJD plays a role in the treatment of cerebral ischemic injury through anti-inflammation. Therefore, animal experiments were designed to verify our hypothesis.

### 3.4. HLJD Aqueous Extract Content Detection

**Figure 5:**
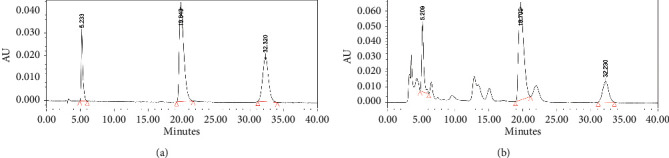
High-performance liquid chromatography; (a) reference substance; (b) sample; 1, 2, and 3 were geniposide, baicalin, and berberine hydrochloride, respectively.

Determination of baicalin, berberine hydrochloride, and geniposide in HLJD is shown in Figure 5.

### 3.5. HLJD Alleviated the Degree of Brain Injury in MCAO Rats

**Figure 6:**
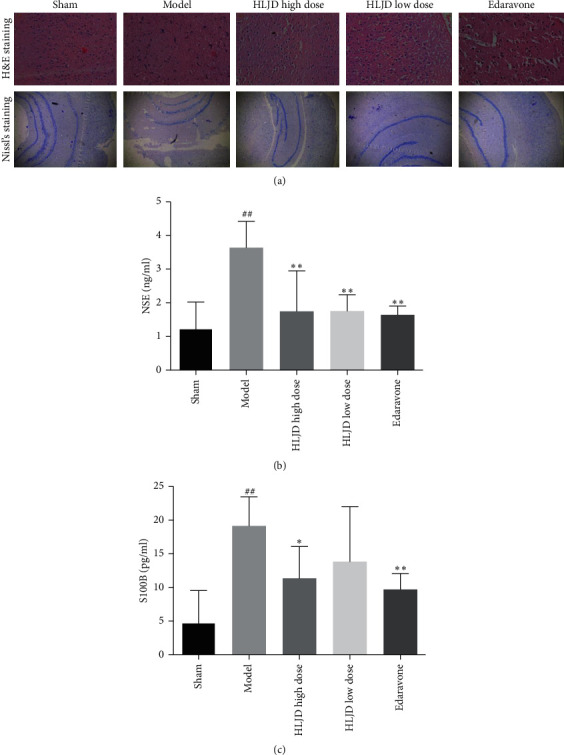
(a) Histopathological sections of MCAO rats by H&E staining and Nissl body staining; (b) NSE and S100 B sera contents of MCAO rats; compared with the sham operation group, ^##^*p* < 0.01, ^#^*p* < 0.05; compared with the model group, ^*∗∗*^*p* < 0.01, ^*∗*^*p* < 0.05.

In the sham operation group, the structure of the cortex and hippocampus was complete, the neurons were compact and their level was clear, the cytoplasm was rich, the nuclei were stained in the middle, and their morphology was normal. The vascular endothelial cells were neat and tightly connected, and there were no congestion and blood cell occlusion. The perivascular tissue was tight. In the model group, the focal area was obviously edematous, the staining clearly reduced, the arrangement of neurons was disordered with presence of several deformations and necrosis, loose density, unclear nuclei, loose deep staining, cell bodies contracted, cell membranes and surrounding boundaries were obvious, and interstitium loose and sieve. The loss of neurons and the proliferation of glial cells were obvious. The vascular endothelial cells were swollen, the vessels wall deformed, and the perivascular space enlarged. In HLJD high-dose group, HLJD low-dose group, and edaravone group, the degree of degeneration and necrosis decreased when compared with the model group (Figure 6(a)).

In the sham operation group, the neurons in the cortex and hippocampus were well defined and the cytoplasm were covered with dark blue granular Nissl bodies, especially in the periphery of the cytoplasm. In the model group, the outline of neurons was blurred with some cytoplasm autolysis, particle loss, uneven staining, nucleus fragmentation, and dissipation. The Nissl body content significantly decreased, and the layout was unclear. Compared with the model group, the morphological changes of Nissl bodies in the HLJD high-dose group, HLJD low-dose group, and edaravone group decreased in varying degrees and the cytoplasm became darker (Figure 6(a)).

The expressions of NSE and S100 B were low in the sham operation group, and NSE and S100 B contents in the model group were significantly higher than those in the sham operation group (*p* < 0.01). Compared with the model group, the HLJD high-dose group, HLJD low-dose group, and edaravone group could reduce NSE and S100 B in sera content of MCAO rats. There was significant difference between the HLJD high-dose group, the edaravone group, and the model group (*p* < 0.01 or *p* < 0.05) (Figure 6(b)).

### 3.6. HLJD Downregulated the Expression of Inflammatory Cytokines in the Brain of MCAO Rats

**Figure 7:**
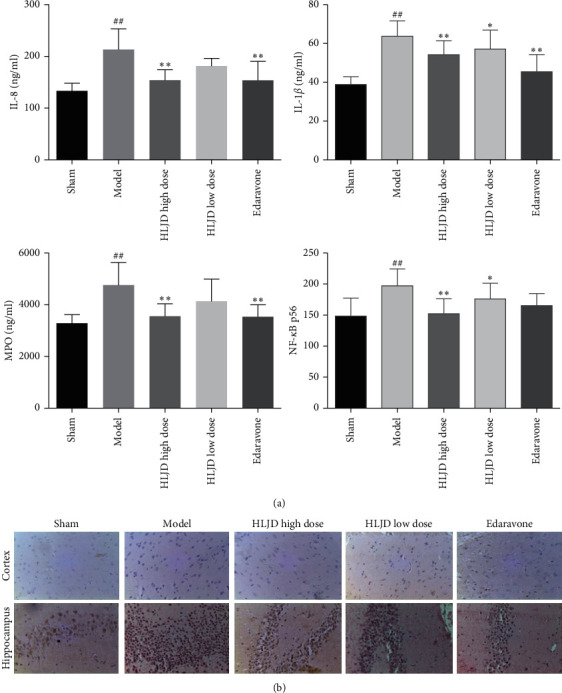
(a) IL-1*β*, IL-8, MPO, and NF-*κ*Bp65 contents in MCAO rats' brains; (b) distribution of NF-*κ*Bp65 in MCAO rats' hippocampi and cortices; compared with the sham operation group, ^##^*p* < 0.01, ^#^*p* < 0.05; compared with the model group, ^*∗∗*^*p* < 0.01, ^*∗*^*p* < 0.05.

Interleukin-1 (IL-1*β*), neutrophilic chemokines (IL-8), and myeloperoxidase (MPO) in the affected side of the brain tissue in the model group were significantly higher than those in the sham operation group (*p* < 0.01). IL-1*β*, IL-8, and MPO in the brain tissue of the HLJD high-dose group and edaravone group were significantly lower than those of the model group (*p* < 0.01). IL-1*β*, IL-8, and MPO in the brain tissue of the affected side were also decreased in the HLJD low-dose group (Figure 7(a)).

In the sham operation group, most of the NF-*κ*B immunoreactive positivity was observed in the cytoplasm. The NF-*κ*B immunoreactive cells in the ischemic cerebral cortex of MCAO rats had an obvious nuclear translocation, brown deposits were mostly expressed in the nucleus, and most of the neurons were degenerative and necrotic. After 2 h of ischemia and 22 h of reperfusion, the positive expression of NF-*κ*Bp65 in the cerebral cortex and hippocampal CA1 and CA3 areas of rats was significantly increased. The HLJD high-dose group, HLJD low-dose group, and edaravone group could obviously downregulated the expression of NF-*κ*Bp65 protein in the cortex, CAI, and CA3 of MCAO rats, which were significantly different from that in the model group (*p* < 0.05) (Figure 7(b)). The results of ELISA and immunohistochemistry confirmed that HLJD could reduce the expression of inflammatory cytokines in the brain of MCAO rats and play a role in protecting the brain from injury.

### 3.7. HLJD Regulated MCAO Rats' Inflammatory Damage through Regulating the 5-LOX Pathway

**Figure 8:**
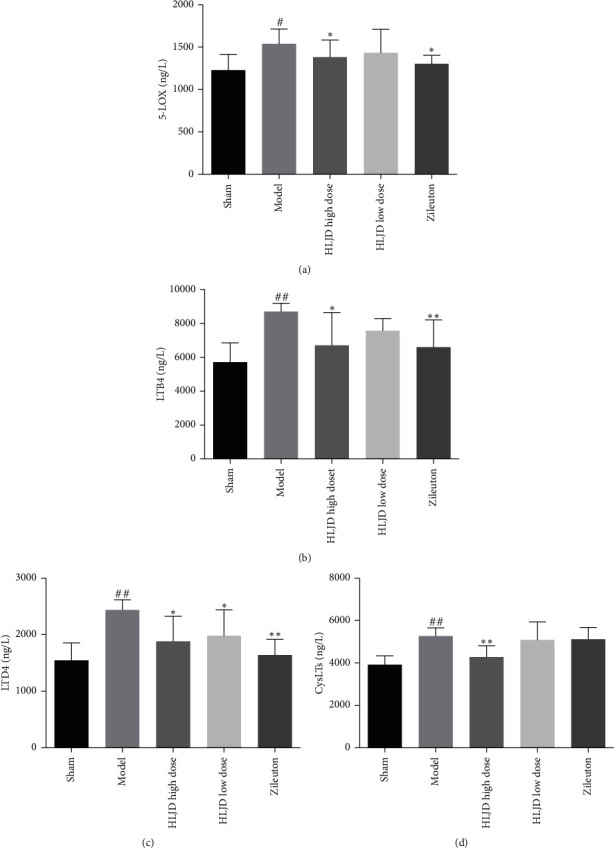
Expressions of 5-LOX, LTB4, LTD4, and CysLTs in MCAO rats' brains were detected by ELISA; compared with the sham operation group, ^##^*p* < 0.01, ^#^*p* < 0.05; compared with the model group, ^*∗∗*^*p* < 0.01, ^*∗*^*p* < 0.05.

ELISA results showed that the expressions of 5-LOX, LTD4, LTB4, and CysLTs in the brain tissues of MCAO rats were significantly increased (*p* < 0.01, Figure 8). After treatment with HLJD and the 5-LOX inhibitor, Qiliudong, the expressions of 5-LOX, LTD4, LTB4, and CysLTs significantly decreased when compared with the model group (*p* < 0.05, Figure 8). We found that the treatment effect of HLJD was better in the high-dose group.

**Figure 9:**
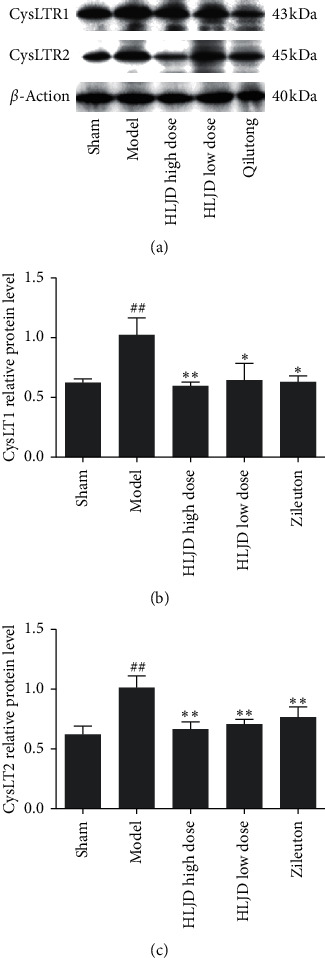
Protein levels of CD11b and CD206 were tested through western blot; compared with the sham operation group, ^##^*p* < 0.01, ^#^*p* < 0.05; compared with the model group, ^*∗∗*^*p* < 0.01, ^*∗*^*p* < 0.05.

We detected the expression of CysLT1 and CysLT2 in the two receptors of CysLTs by WB, and the results showed that CysLT1 and CysLT2 expressions were significantly increased in MCAO rats and that CysLT1 and CysLT2 expressions could be significantly downregulated after treatment with HLJD and Qiliudong when compared with the model group (*p* < 0.05, Figure 9). The results were consistent with ELISA results.

## 4. Discussion

**Figure 10:**
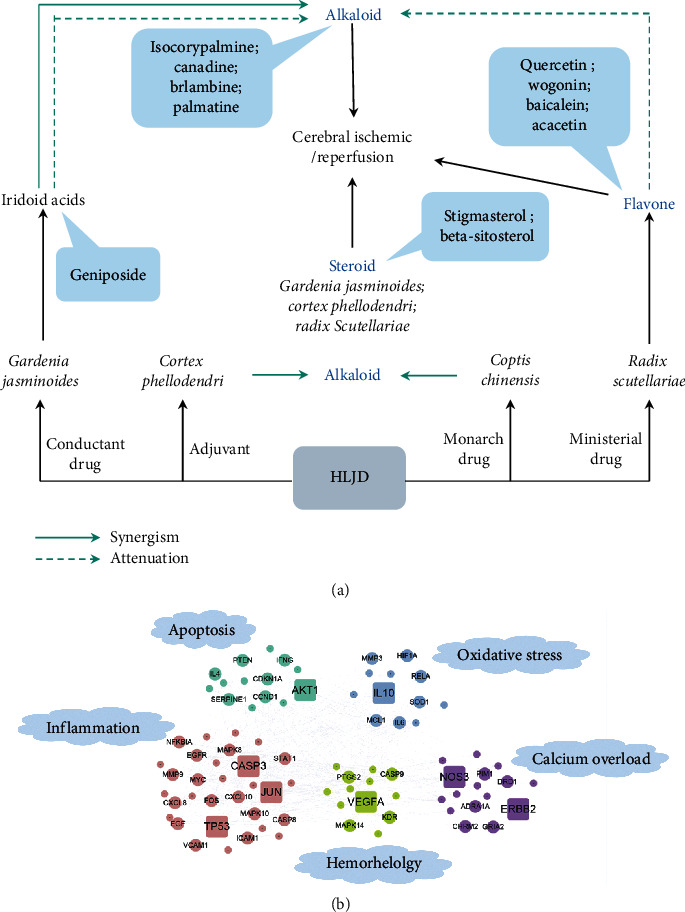
(a) HLJD material base diagram; (b) molecular mechanism of HLJD against cerebral ischemia.

*Huanglian* is the main toxic substance in HLJD, and the toxicity of *Huangbo* is weak. *Huangqin* and *Zhizi* have no obvious toxicity, among which *Huangqin* has detoxification effect in HLJD [18, 19]. Alkaloids are the main active components and have obvious anti-inflammatory effects, but their oral utilization is low and their toxicity and side effects are also great. In the process of decoction, *Huanglian* and *Huangbo* were used to improve the efficacy of alkaloids. At the same time, the flavonoids (quercetin, wogonin, baicalein, and acacetin) in *Huangqin* precipitated and reduced the toxicity of alkaloids. The iridoid acids (geniposide) in *Zhizi* and alkaloids also precipitate, which reduces the toxic and side effects of alkaloids but improves their efficacy [20–23]. 13 chemical constituents in HLJD are shown to be common to the 4 drugs and are the main effective components for the treatment of cerebral ischemia, and they also improve the efficacy of alkaloids and other components, including alkaloids, flavonoids, and steroids (Figure 10(a)). It also reflects the superposition of quantity. In this paper, there was no further study on the material basis of HLJD, which can be verified by cell experiments in the later stage.

Through the analysis of network pharmacological results, it is known that the main components of anticerebral ischemia in HLJD include alkaloids (isocorypalmine, canadine, brlambine, and palmatine), steroids (stigmasterol and beta-sitosterol), and flavonoids (quercetin, wogonin, baicalein, and acacetin). Flavonoids have anti-inflammatory effects by activating NF-*κ*B and inhibiting the expression of different proinflammatory cytokines/chemokines, including tumor necrosis factor-*α* (TNF-*α*), IL-1*β*, interleukin-6 (IL-6), and IL-8 [24]. Steroids can reduce the occurrence and development of brain edema, improve the function of blood-brain barrier, and reduce protein leakage in response to acute cerebral ischemia [25]. In the GO functional enrichment analysis, it involved the response to ketones and steroids. It was further verified that flavonoids and steroids were involved in the treatment of cerebral ischemia. In summary, the effect of HLJD in vivo is the result of drug synergy and antagonistic detoxification.

Through the topological analysis of the “component-target-disease” network diagram, the main components and targets of anticerebral ischemia in HLJD were identified. GO enrichment analysis and KEGG pathway enrichment analysis showed that it involved several cancers and inflammation-related pathways, and many of which were related to cerebral ischemia. According to the enrichment results, the molecular mechanism of HLJD in cerebral ischemia was predicted as shown in Figure 10(b). It mainly involves apoptosis, inflammation, oxidative stress, Ca^2+^ overload, and hemorheology. According to the network pharmacological prediction results, anticerebral ischemia is mainly related to inflammation and HLJD is a classical prescription for clearing heat and detoxification. The anti-inflammatory components of HLJD were docked with the key inflammatory protein COX-2 and 5-LOX, and it was found that the components with anti-inflammatory effects could act on these molecules. Based on this, we designed animal experiments to verify the results of the network pharmacology and molecular docking prediction.

Previous studies have shown that oxygen supply and neuroinflammation are the main causes of cell death and brain edema in the development of cerebral ischemia [26]. It is reported that the levels of NSE and S100 B increase in patients with brain and nerve injuries [27]; therefore, NSE and S100 B are considered to be biomarkers of central nervous system injury [28]. Through pathological section staining and detection of NSE and S100 B in serum, we found that HLJD could improve the degree of brain injury in MCAO rats.

NF-*κ*B is an important transcriptional regulator of inflammation, which is widely associated with ischemia and other inflammatory diseases [29–31]. The NF-*κ*Bp65/p50 heterodimer complex and its inhibitor I*κ*B*α* are inactive in the cytoplasm under normal conditions. I*κ*B kinase *β* is rapidly phosphorylated and activated after cerebral ischemia-reperfusion injury and subsequently induces I*κ*B*α* phosphorylation and degradation. Therefore, NF-*κ*Bp65/p50 dimer is released and transported to the nucleus, which leads to the release of a variety of proinflammatory factors, such as TNF-*α* and interleukins [32]. TNF-*α* and IL-1*β* mediate the activation of the cytoplasmic NF-*κ*B which translocate into the nucleus, where it further stimulates the transcription and expression of proinflammatory cytokines and other markers such as COX-2 and aggravating the inflammatory response [33]. NF-*κ*B also drives microglial morphological activation and microglial NF-*κ*B activation in brain ischemia appears to be largely neurotoxic [34]. Constitutive activation of I*κ*B kinase to promote nuclear translocation of NF-*κ*B increased infarct size [35]. Neuronal necrosis was observed in the cortex and hippocampus of rats in the model group, and the content of NF-*κ*Bp65 was increased in the nucleus. HLJD in the treatment group could significantly downregulate the expression of NF-*κ*Bp65 in the hippocampus and cortex of MCAO rats.

Leukotriene (LTs) is a short-term but effective proinflammatory lipid medium, which is produced by the arachidonic acid and catalyzed by 5-LOX [36]. The 5-LOX metabolism of the arachidonic acid requires the 5-LOX activating protein (FLAP) to form two groups of leukotrienes: LTB4 that is formed by the LTA4 hydrolase (LTA4H) and CysLTs (including LTC4, LTD4, and LTE4) that is formed by the LTC4 synthase. Each group acted on its own specific receptors (BLT and CysLT receptors, respectively) [37]. CysLTs is involved in leukocytes' aggregation, adhesion of microvascular endothelial cells, and production of oxygen free radicals; therefore, the accumulation of CysLTs in the brain plays a key role in cerebral ischemia [38–41]. After cerebral ischemic injury, CysLTs' synthesis and release are increased, which increases the expression of inflammatory molecules, leading to brain edema, neurological impairment, and secondary brain injury [42, 43]. LTB4 has been shown to activate leukocytes [44] and T cells [45] and to stimulate the production of cytokines and NF-*κ*B [46]. LTB4 increased production during the acute stages of ischemic stroke can worsen the extent of tissue infarct [47]. At present, LTB4 role in cerebrovascular diseases mainly focuses on its chemotaxis effect on neutrophils. The Barone study [48] showed that a large number of neutrophils gathered in the cerebral infarcted tissues of MCAO rats and that the increase in the binding amount of LTB4 and its receptor in these tissues was consistent with the expression of myeloperoxidase (MPO), a marker of neutrophil infiltration. Meanwhile, studies have shown that the expression of LTB4 and its receptor can be detected in neutrophils [49, 50]. The 5-LOX inhibitor, Qiliudong, can reduce the activity of MPO and the expression of NF-*κ*B in the brain of MCAO rats, which alleviates the brain injury and inflammation in MCAO rats [51].

**Figure 11:**
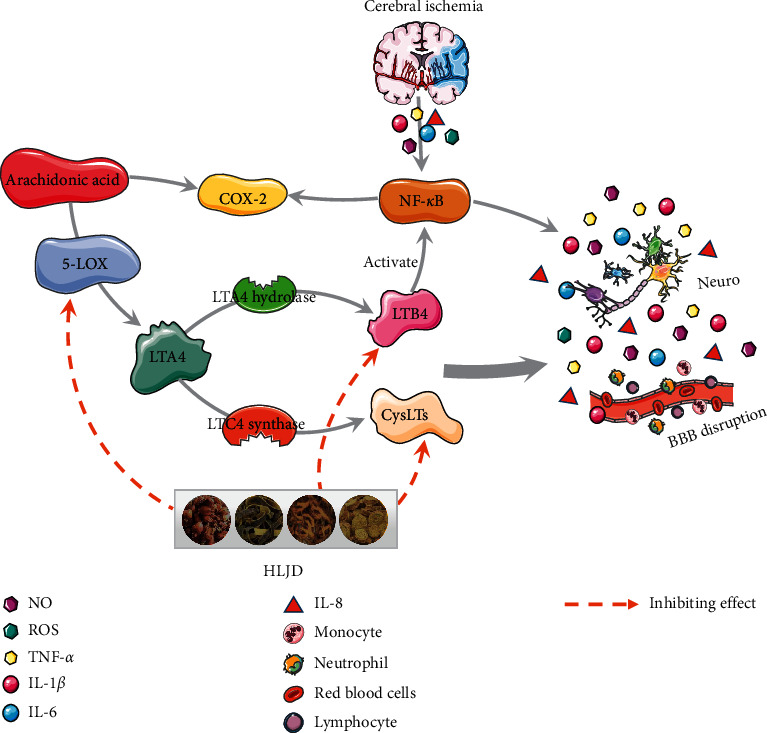
Mechanism of protective effect of HLJD on inflammatory injury induced by cerebral ischemia.

In this part of the study, the expressions of CysLTs and LTB4 in the 5-LOX pathway were detected by ELISA, and the protein expression of the CysLTs receptors, Cyslt1 and Cyslt2, were detected by western blot. We found that HLJD inhibits the 5-LOX pathway. Therefore, HLJD may inhibit the proinflammatory effect by downregulating the 5-LOX pathway, thus playing a protective role in cerebral ischemia injury (Figure 11).

## 5. Conclusions

We predicted the material basis and molecular mechanism of HLJD in the treatment of cerebral ischemia through network pharmacology and molecular docking and clarified that the anticerebral ischemia effect of HLJD is the result of drug synergism and antagonistic detoxification. Through the establishment of the MCAO rat model, it was confirmed that HLJD reduces the expression of neuronal injury and inflammatory factors following cerebral ischemia by downregulating the 5-LOX pathway. In summary, the integrated strategy that was developed in our study provided novel insights into the therapeutic potential and mechanisms of traditional Chinese medicine, HLJD.

## Figures and Tables

**Table 1:** Docking information.

Docking with the COX-2			Docking with the 5-LOX		
Molecule name	Gibbs free energy kJ/mol	Ki *μ*mol/L	Molecule name	Gibbs free energy kJ/mol	Ki *μ*mol/L
Acacetin	−7.34	4.14	Acacetin	−6.40	20.37
Baicalein	−5.11	179.50	Baicalein	−4.25	761.47
Berberine	−8.25	0.90	Berberine	−6.54	16.10
Beta-sitosterol	−7.91	1.60	Beta-sitosterol	−7.72	2.18
Campesterol	−8.63	0.47	Campesterol	−7.92	1.56
Cavidine	−9.43	0.12	Cavidine	−6.64	13.67
Chelerythrine	−8.67	0.44	Chelerythrine	−6.64	13.46
Coptisine	−8.25	0.90	Coptisine	−6.72	11.89
Crocetin	−7.30	4.48	Crocetin	−6.47	18.20
Epiberberine	−9.30	0.15	Epiberberine	−7.31	4.39
Isoimperatorin	−7.67	2.38	Isoimperatorin	−5.84	52.80
Kaempferol	−6.87	9.21	Kaempferol	−5.66	71.10
Obacunone	−7.70	2.28	Obacunone	−8.28	0.85
Oroxylin A	−7.07	6.61	Oroxylin A	−6.61	14.31
Palmatine	−7.38	3.87	Palmatine	−6.21	28.20
Quercetin	−7.05	6.76	Quercetin	−5.36	118.60
Rutaecarpine	−8.49	0.60	Rutaecarpine	−7.88	1.67
Sitosterol	−10.49	0.20	Sitosterol	−8.35	0.74
Skullcapflavone II	−5.60	79.05	Skullcapflavone II	−6.09	34.51
Stigmasterol	−9.45	0.12	Stigmasterol	−7.98	1.42
Wogonin	−7.23	5.00	Wogonin	−5.69	67.04
NS-398	−8.01	1.34	Aa-861	−7.86	1.72

## Data Availability

The data used to support the findings of this study are included within the article.
